# Dynamic Changes in Glutenin Macropolymer during Different Dough Mixing and Resting Processes

**DOI:** 10.3390/molecules26030541

**Published:** 2021-01-21

**Authors:** Yulin Feng, Huijuan Zhang, Jing Wang, Haitao Chen

**Affiliations:** China-Canada Joint Lab of Food Nutrition and Health (Beijing), Beijing Technology & Business University, Beijing 100048, China; fengyl983717@163.com (Y.F.); zhanghuijuan@th.btbu.edu.cn (H.Z.); wangjing@th.btbu.edu.cn (J.W.)

**Keywords:** glutenin macropolymer (GMP), dynamic rheological, disulfide bond, secondary structure, microstructure

## Abstract

The glutenin macropolymer (GMP), which is an important component of the glutenin protein in wheat flour, plays a prominent role in governing dough properties and breadmaking quality. This study investigated the changes in GMP properties during the mixing and resting stages of dough processing. The results show that the GMP content decreases by about 20.20% when the mixing time increases from 3 to 5 min, while increasing the resting time can lead to restoration of some GMP contents. Resting promotes greater formation of large-sized GMP particles, which is likely related to the increased disulfide bond content in the GMP during this process. In contrast, the mechanical force of mixing causes GMP depolymerization and formation of smaller particles. Furthermore, after mixing, the protein secondary structure tends to be disordered, the protein morphology becomes irregular, and the protein subunit ratio changes. Thus, mixing has many of the opposite effects to resting, although resting can (to some extent) restore the properties of the GMP after mixing. However, excessive resting time can lead to negative results, reflected in lower disulfide bond (SS) and GMP contents, and more irregular particle sizes. The presented results suggest that dough mixing induces rearrangement of the dough’s protein structure, and resting somewhat restores the chemical bonds and internal protein structure.

## 1. Introduction

Proteins markedly affect the quality of wheat flour products [1,2]. The gluten protein, which is the primary determinant of functional or technological properties of wheat flour, contains glutenin and gliadin and affects the quality of wheat products and the rheology of the dough [3,4]. The glutenin macropolymer (GMP) is an important component of the glutenin protein that contains a high-molecular weight glutenin subunit (HMW-GS) and a low-molecular weight glutenin subunit (LMW-GS). Additionally, the GMP is insoluble in 1.5% (*w*/*v*) sodium dodecyl sulfonate (SDS) solution, allowing for its isolation as a gel layer [5,6,7].

Several studies have investigated the influence of the quantity, rheology, and structural characteristics of the GMP on its breadmaking capabilities based on assessing dough properties [8,9,10]. The GMP has a direct relationship with pasta processing parameters; additionally, loaf volume is positively correlated with the GMP elastic modulus (G′) [11]. Studies of dough properties during the production of Chinese steamed bread (CSB) showed that the GMP wet weight decreased sequentially during mixing, fermentation, and remixing of CSB dough, and then increased significantly (*p* < 0.05) during the resting stage [12].

Dough processing, including the mixing and resting steps, affects the formation of the glutenin network and GMP polymerization. Several studies have described gluten polymerization during product generation [11,13,14]. One report showed that disulfide bonds (SSs) are formed in the pre-existing gluten protein network of fresh pasta during cooking, and glutenin polymerization occurs faster than gliadin–glutenin copolymerization [13]. Another study determined that dough mixing reduces the average size of GMP particles in flour [10]. Similarly, analysis of samples extracted from the mix, compound, and sheets of dough revealed that the GMP weight decreases with each step of alkaline noodle processing [15]. Additionally, evaluating glutenin particle breakdown and re-assembly as a function of various mechanical mixing regimes showed a relationship between glutenin particle properties and aspects of the resulting dough [16]. The type of shear cell applied during dough processing is critical, so different types of shear must be considered when designing new dough processing equipment [17].

In addition, some studies also involve other changes in the GMP during processing. Veraverbeke et al. explored the levels and composition of SDS-unextractable glutenin polymers during mixing and fermentation [18]. Skerritt et al. pointed out that the loss of the HMW-GS may cause molecular weight distribution and the composition of the disulfide-bonded GMP [19]. The study of Don et al. proposed that the particle properties of the GMP affected by mixing could influence gluten function in dough [16]. Another research showed that the changes in size distributions of the protein and the glutenin subunit composition within SDS-unextractable polymers indicated that some HMW-GSs play a major role in forming the backbone of SDS-unextractable polymers [1]. Most of these studies focused on the effect of mixing on the SDS extractability of the GMP and the roles of different glutenin subunits. However, other GMP properties such as the changes in chemical bonds, protein secondary structure, and network microstructure during mixing and resting are rarely reported, which need further study.

This study aims to provide further insight into GMP behavior during dough mixing and resting. The changes to GMP content, rheology, particle size, and SSs are analyzed, and reversed-phase high-performance liquid chromatography (RP-HPLC) is applied to examine the ratio of GMP subunits. The GMP microstructure and dispersion are observed at each stage using scanning electron microscopy (SEM) and confocal laser scanning microscopy (CLSM), in order to visualize the formation of the protein network and the shape of the GMP protein. These analyses improve our understanding of GMP (de)polymerization mechanisms during dough mixing and resting and provide a theoretical basis for research aimed at regulating the quality of wheat flour products.

## 2. Results and Discussion

### 2.1. GMP Content

**Figure 1:**
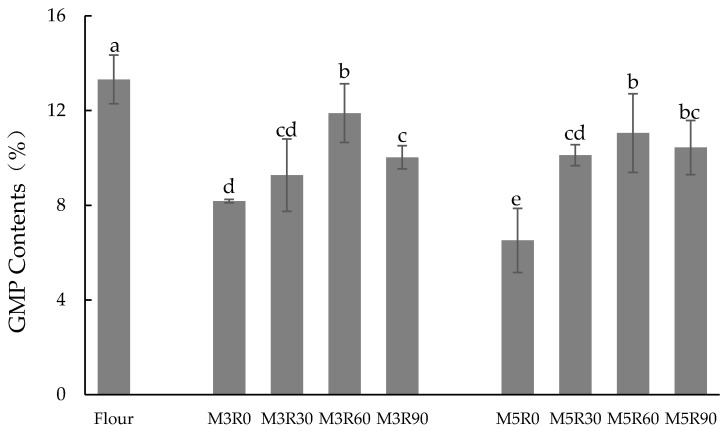
Changes in glutenin macropolymer (GMP) contents (%, expressed as percent GMP in crude protein content of flour). Flour: wheat flour without mixing or resting. M3R0–M3R90: mixing for 3 min and resting for 0, 30, 60, and 90 min, respectively. M5R0–M5R90: mixing for 5 min and resting for 0, 30, 60, and 90 min, respectively. Data are mean values ± standard variation of three replications and different letters indicate a significant difference at *p* < 0.05.

The content and quality of the GMP plays an extremely important role in the baking quality of dough, thereby affecting properties such as the viscoelasticity of the dough and the volume of the resulting bread [2,20]. The GMP contents in the flour (before dough processing) and in the dough following different mixing and resting times are shown in Figure 1. The flour group had the highest GMP content (13.31%), and the presented dough mixing and resting stages displayed a significantly lower (*p* < 0.05) GMP content. Considering the M3R0 and M5R0 groups, the GMP content in the dough decreased significantly (*p* < 0.05), from 8.17% to 6.52%, as the mixing time increased, suggesting the protein macropolymer was converted into smaller, SDS-soluble polymers. The result is in agreement with previous research, indicating the increasing extractability of glutenin polymers in the mixing process [1,19]. Weegels et al. also reported that extractability of flour proteins would increase when mixing flour with water into a dough [21]. The reduced GMP content after a longer dough mixing process is consistent with the concept that depolymerization of the GMP occurs as a result of the mechanical agitation [16]. Except for the breaking of some chemical bonds [19], the loss of some HMW-GSs existing as the protein backbone during mixing may also be the cause of the decrease in the GMP content [1].

Figure 1 clearly shows that the resting process increases the GMP content. The GMP content reached a peak value when the allowed resting time was 60 min. Following the dough mixing process, after the dough is allowed to rest for a period of time, the network structure in the dough can potentially automatically adjust, and the SS content in the dough increases. Therefore, in this resting stage, the depolymerized glutenin macromer re-crosslinked via disulfide bonds between the subunits, and the GMP content increased. Furthermore, the GMP content was higher in the flour compared to any of the processed doughs. It seems that in spite of some restoration of the GMP content level during resting, it cannot recover to the status before dough processing. The proposed depolymerization and repolymerization mechanisms may provide the explanation to the results [1].

When the dough resting time was longer than 60 min, the GMP content displayed a downward trend. We propose that the protease in the wheat flour was activated by sulfur-containing amino acids, such as cysteine [22]. After such modifications of the internal structure of the dough, some glutenin aggregates decomposed, which led to the observed reduction in GMP content with resting times longer than 60 min [23]. In the investigation of Morel et al., the authors also found that proteases and temperature affected the stability of flour samples in distribution and extractability of total wheat flour proteins using size-exclusion high-performance liquid chromatography [24].

### 2.2. Dynamic Rheological Properties of GMP

**Figure 2:**
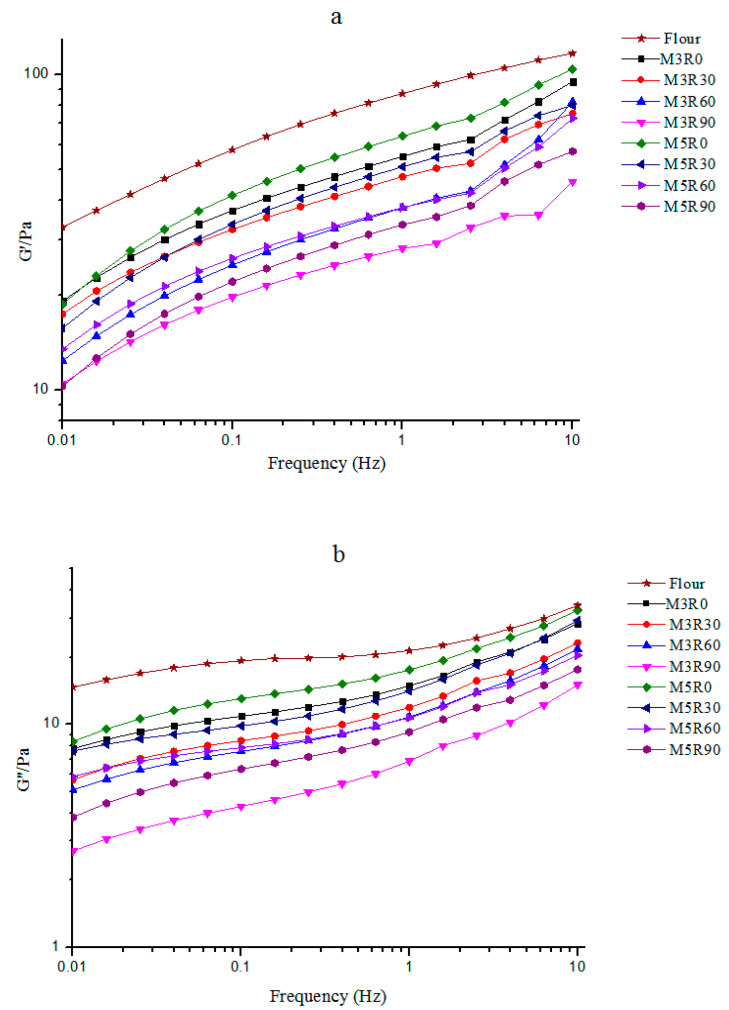
Changes in viscoelastic modulus G′ (**a**) and G′′ (**b**) for the GMP. Flour: wheat flour without mixing or resting. M3R0–M3R90: mixing for 3 min and resting for 0, 30, 60, and 90 min, respectively. M5R0–M5R90: mixing for 5 min and resting for 0, 30, 60, and 90 min, respectively.

The rheological properties of the GMP are important indicators of GMP quality and gluten characteristics because they reflect gluten strength and dough viscoelasticity, which significantly affect the quality of baked products [10,16]. The dynamic rheology of the GMP with different dough mixing and resting times is shown in Figure 2. The trends observed for the GMP protein elasticity modulus (G′) and viscosity modulus (G″) values for each group were consistent; further, they all increased with the higher scanning frequency throughout the linear region. The G’ values were greater than G”, indicating that the extracted GMP embodied properties similar to solids. The viscoelastic moduli of GMP proteins in the mixed dough groups were lower than the flour group, and extended mixing times increased the viscoelastic moduli of the GMP, likely because of the de-polymerization of the GMP molecules as a result of the applied mechanical force. However, as the resting time increased over the scanning range, the viscoelastic moduli of GMP proteins decreased, which may be a result of the repolymerization of GMP molecules. Additionally, it was reported [16] that prolongation of the resting time (i) allows for re-polymerization of the GMP molecules destroyed by mechanical force during the mixing process and (ii) leads to a decrease in the GMP viscoelastic moduli. Similarly, dynamic rheological changes to the GMP during the production of Chinese steam bread demonstrated that gluten polymerization decreased the G′ and G′′ values of the GMP in the system, while the fermentation and remixing processes induced a slight recovery of the G′ and G′′ values of the GMP [12].

### 2.3. SH/SS Exchange and SH Oxidation

As presented in Table 1, the accessible thiol (SH_free_) content in the GMP obtained after the dough resting processes was reduced compared with the flour group. Slight reduction (with no significant differences) at the end of mixing was also observed. It was previously determined that the mixing process decreased the SH_free_ content of CSB dough, which may be related to the formation of a structure caused by mechanical deformation in which SH_free_ was buried within inaccessible structural regions in proteins [12]. We found that the resting process also decreased the SH_free_ content of GMP proteins, and a greater decrease in SH_free_ content was observed with increasing resting time. This is likely because some of them participated in the formation of disulfide bonds (SSs) during resting.

A relatively short resting process (30–60 min) significantly (*p* < 0.05) increased the total thiol equivalent groups (SH_eq_) and SS contents in the GMP (except for the M3R90 and M5R90 groups). This is because exposure to oxygen during the resting process resulted in oxidation of some SH_free_ in the GMP to form SSs, thereby increasing the content of SSs in the dough [25]. Additionally, the resting process enabled further formation of the gluten network and allowed the glutenin subunits to bind together more tightly, which promoted conversion of the thiol groups between polypeptide chains into disulfide bonds [26].

However, more prolonged resting times (e.g., in the M3R90 and M5R90 groups) led to decreased SS content in the GMP. This is likely because, over time, microbes and protease enzymes cause partial GMP disintegration via intramolecular and intermolecular disulfide bond cleavage. Additionally, extremely long resting times cause the GMP peptide chains to expand more, and secondary bonds, such as the disulfide bonds between the chains, are broken [23,27]. We observed that the SS content was positively correlated with the GMP content (r = 0.800, *p* = 0.017), which supports the relationship between the changes in GMP and SS contents during the dough mixing and resting processes.

### 2.4. Particle Size Analysis of GMP

**Figure 3:**
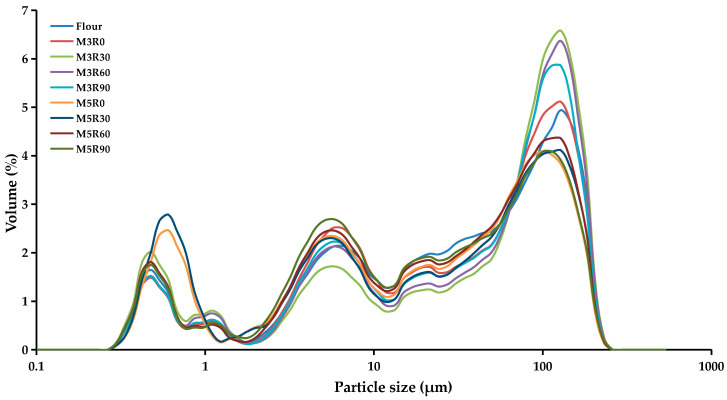
Particle size analysis of the GMP. Flour: wheat flour without mixing or resting. M3R0–M3R90: mixing for 3 min and resting for 0, 30, 60, and 90 min, respectively. M5R0–M5R90: mixing for 5 min and resting for 0, 30, 60, and 90 min, respectively.

GMP gel is known to exist as sphere-like granules [16]; therefore, the diffraction principle of the laser particle size analyzer was used to determine the effects of different mixing and resting times on GMP particle sizes. Figure 3 shows that the particle size distribution of the GMP among the collected samples was between 0.296 and 231 μm. The GMP particle size distribution was divided into three different ranges: small (0–11 μm), medium (11–50 μm), and large (50–231 μm). The volume distribution of the GMP particle size is shown in Table 2. Relative to the flour group, the mixing and resting processes significantly (*p* < 0.05) changed the GMP particle sizes in each distribution range. For example, 30 and 60 min resting times increased the content of the large-particle size GMP (50–231 μm). In contrast, increasing the mixing time (from 3 to 5 min) significantly increased the small-particle size volume fractions of the GMP. It was previously reported [16] that the mechanical force of the mixing process depolymerizes the GMP in the dough, and during the dough resting process, the partially depolymerized glutenin polymer re-polymerizes, resulting in the ultimate formation of larger-sized GMP particles.

With the increasing resting time, the large-size GMP content first increased and then decreased, thus exhibiting a similar trend to the sulfhydryl and disulfide bond contents of the GMP. Therefore, the changes in the SS content of the GMP may be the reason for the conversion of particle size fractions in different ranges. When the dough had completely rested, the GMP also interacted with the oxygen in the air as well as the microorganisms and proteases in the dough. These interactions interrupt the secondary bonds (i.e., disulfide bonds between the chains), which disconnects the subunits, resulting in a reduction in the large-particle size GMP content [23,26].

### 2.5. Secondary Structure of GMP

The characteristic protein absorption bands observed in Fourier transform infrared (FTIR) spectroscopy include the amide I region (1600–1700 cm^−1^) and the amide II region (1500–1600 cm^−1^). The absorption band in the amide I region is generated by C=O bond stretching vibrations, and the absorption band in the amide II region is generated by the bending vibration of the N-H bond [28]. Therefore, analysis of the amide I region can provide information about the secondary structure of the protein. The secondary structure of most proteins and their corresponding wavelength intervals can be classified as α-helix (1650–1660 cm^−1^), β-sheet (1610–1640 cm^−1^), β-turn (1660–1700 cm^−1^), or random-coil (1640–1650 cm^−1^) structures [29]. Baseline correction, Gaussian deconvolution, and second-order derivation of the amide I region were performed for the GMP sample spectra to quantitatively analyze the effects of different mixing and resting times on the secondary structure of the GMP. The amount of each secondary structure present in each sample was expressed as a percentage, calculated by considering the relevant peak area as a fraction of the total peak area, and the results are provided in Table 3.

The FTIR results indicate that the β-sheet structure was the main secondary structure in all GMP samples. Previous studies have also shown that β-sheet is the main type of secondary structure in the gluten protein [30,31,32]. Furthermore, it was reported [33] that the increased α-helix content corresponds to a more ordered structure, and the structural stability of a gluten protein was positively correlated with the β-sheet content. In this study, samples that experienced the same mixing time showed an upward trend in the β-sheet and α-helical structural contents and a downward trend in the content of the random-coil structure of the GMP with increasing resting times. These results indicate that the protein structure of the GMP tended to become more stable and ordered over longer resting times according to Table 3. These observations were in line with the tendency of the GMP viscoelastic moduli to decrease with prolonged resting times, as determined in the dynamic rheology experiments (see Section 2.2). Previous studies have also reported that the intermolecular disulfide bonds and β-sheet structure act synergistically to stabilize the protein polymers [34,35]. The increasing disulfide bond content during the shorter resting periods (30 or 60 min; Table 1) also illustrates that the protein structure of the GMP became more stable as the resting time was prolonged.

In contrast, increasing the mixing time decreased the α-helical content from 29.71% to 28.58% in GMP proteins, suggesting that the mechanical mixing process reduced the orderliness of the GMP proteins in the dough as presented in Table 3. However, the β-sheet content increased by about 20% as the mixing time was prolonged from 3 to 5 min.

### 2.6. HMW/LMW Ratio of GMP

**Figure 4:**
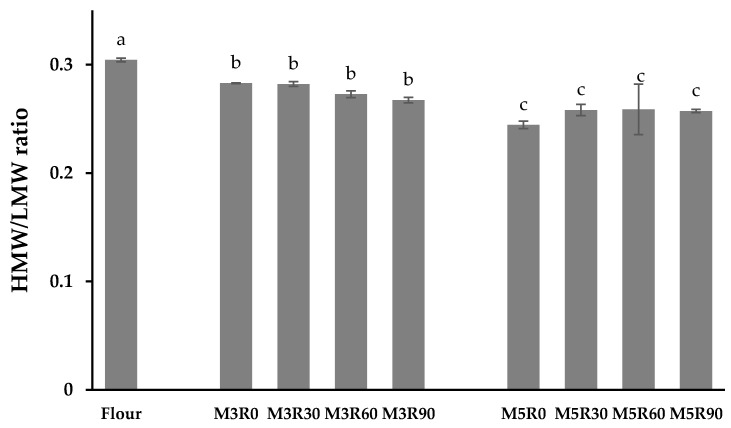
High-molecular weight (HMW)/low-molecular weight (LMW) ratio of the GMP. Data are mean values ± standard variation of three replications and different letters indicate a significant difference at *p* < 0.05. Flour: wheat flour without mixing or resting. M3R0–M3R90: mixing for 3 min and resting for 0, 30, 60, and 90 min, respectively. M5R0–M5R90: mixing for 5 min and resting for 0, 30, 60, and 90 min, respectively.

The HMW-GS and LMW-GS in the GMP form a protein skeleton with a three-dimensional network structure employing disulfide bonds [3]. As the moisture interacts with the flour during the dough preparation, the HMW-GS and LMW-GS form an ordered fibrous macromolecular polymer through the disulfide bonds formed during mechanical agitation, which promotes sulfhydryl/disulfide exchange reactions and polymerization of glutenin subunits into the GMP [36]. Despite its lower content in the GMP relative to the LMW-GS, the HMW-GS has an important effect on gluten protein networks, dough characteristics, and steamed bread quality [37]. With its relatively high content, the LMW-GS contributes to the dough’s rheological properties and high resistance to elongation, as well as the quality of the final dough products [38]. One report demonstrated that the glutenin subunit ratio has a significant effect on the loaf deformation properties and, specifically, that a higher HMW/LMW subunit ratio resulted in a dough with higher resistance during mixing [39]. In this study, RP-HPLC (C8 column) was employed to separate and quantify the GMP subunits. The peak areas were integrated, and the ratio of HMW-GS to LMW-GS was calculated to compare the relative content of each subunit and observe how that ratio changes in response to different processing conditions. This method is effective for studying the changes to the protein subunits, and it has been widely used in various product systems, such as bread [22] and pasta [13]. In this study, the mixing process significantly (*p* < 0.05) decreased the HMW/LMW ratio in the GMP of the dough samples (Figure 4) compared with the flour group, indicating a reduction in the content of the HMW-GS relative to the LMW-GS during the agitation. This observation was in line with a previous study [12], which also reported that the mixing process significantly reduced the content of HMW subunits. This is likely due to the increased hydration and mechanical action during the dough mixing step, which increases the formation of extractable gluten in the dough, while reducing the HMW-GS contents in the GMP that is not extractable from SDS [12]. The study of Skerritt et al. also observed the total amount and proportion of the HMW-GS in the GMP decreased during dough mixing [19]. However, in the report of Aussenac et al., the authors showed the result that the HMW/LMW ratio increased at the initial mixing stage and then decreased after reaching the peak mixing time [1]. Meanwhile, they found that the decline in various HMW-GSs was different during mixing, which indicated that glutenin subunits were released in nonrandom order and the polymers had a hierarchical structure. Therefore, we infer that the contrary may be caused by the various cultivars of the flour used in the studies which resulted in differences in the HMW-GS composition. The composition of the HMW-GS in our study may be easier to be released during mixing and may lead to the decline in the HMW/LMW ratio. This also guides us to incorporate different flour varieties and HMW-GS compositions in further concerned research.

In contrast, the resting process did not significantly affect the HMW/LMW ratio, indicating that the relative contents of HMW and LMW subunits do not change appreciably during this dough processing step. This result is consistant with a previous study [1]. However, the relationship between the HMW/LMW subunit ratio and the GMP content, as a function of different resting times, requires further investigation.

### 2.7. Microstructure of GMP

**Figure 5:**
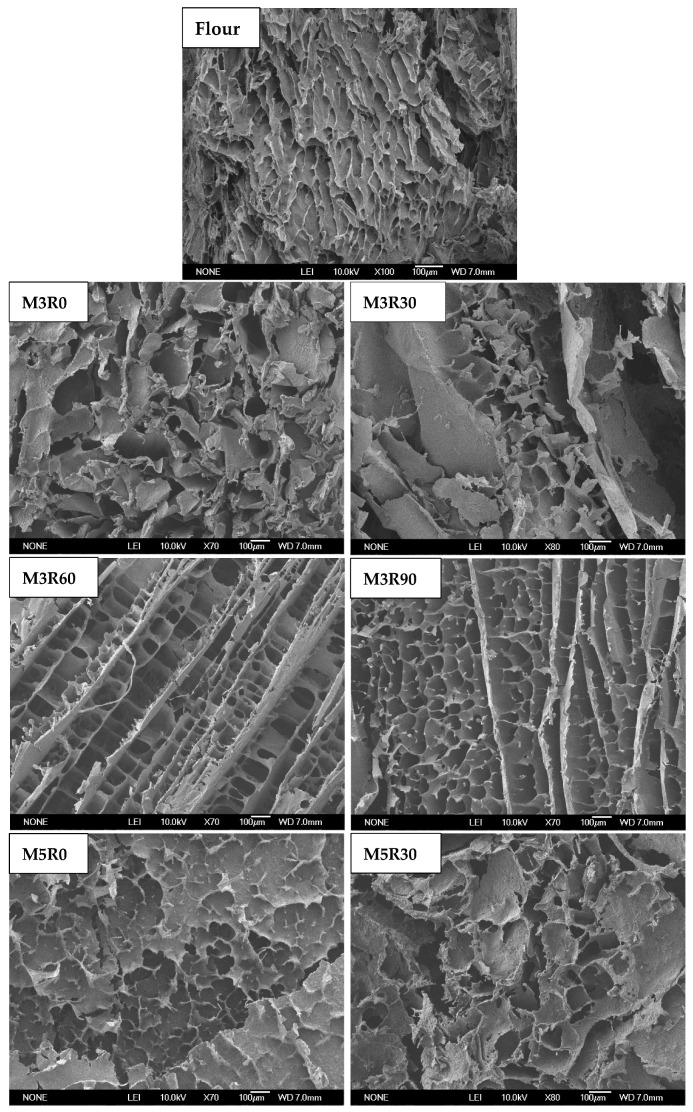

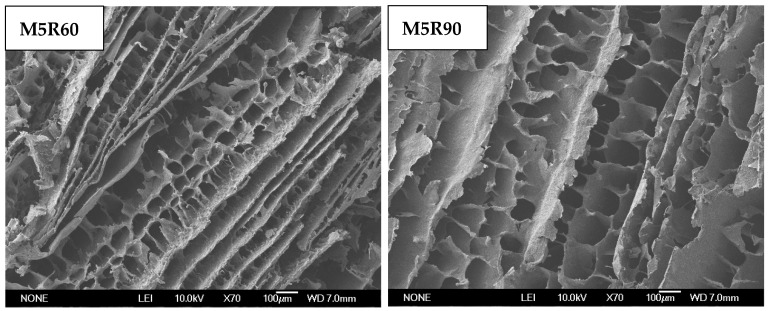
SEM images of the microstructure of the GMP. Flour: wheat flour without mixing or resting. M3R0–M3R90: mixing for 3 min and resting for 0, 30, 60, and 90 min, respectively. M5R0–M5R90: mixing for 5 min and resting for 0, 30, 60, and 90 min, respectively.

The SEM images showing the microstructure of various GMP samples are shown in Figure 5. The microstructure of the GMP exhibits a porous three-dimensional network, although clear differences are observed in the microstructure of the different GMP sample images. The edges of the GMP particles in the flour group were smoother than those in the groups that were mixed and rested, but the flour group also displayed a looser porous structure, poor compactness, and uneven size distributions.

After mixing, the original GMP microstructure was destroyed, and the pore structure became chaotic and disordered. Additionally, the edges were rough and jagged (M3R0 and M5R0 groups, Figure 5). Longer mixing times caused a more severe collapse of the microscopic pore structure, and only the edges of some incomplete holes can be seen. After a certain period of resting time, the GMP microstructure gradually adopted a dense, ordered porous structure, wherein the pores had smooth edges and uniform sizes and distributions (M3R60 and M5R60 groups, Figure 5). These results indicate that the microstructure of the GMP gradually recovers and becomes ordered during the resting process, which is consistent with the experimentally determined SS content results that also suggest increasing network structure formation with longer resting times. The GMP secondary structures presented in Table 3 also show that the resting process promoted enhanced stability and order within the proteins. The microstructures of the GMP samples that were allowed to rest for 90 min exhibited more densely packed, uniformly sized pores within the three-dimensional pore structure, but the edges were no longer smooth, which may indicate that this resting time was too long.

### 2.8. CLSM Characterization of GMP Particles

**Figure 6:**
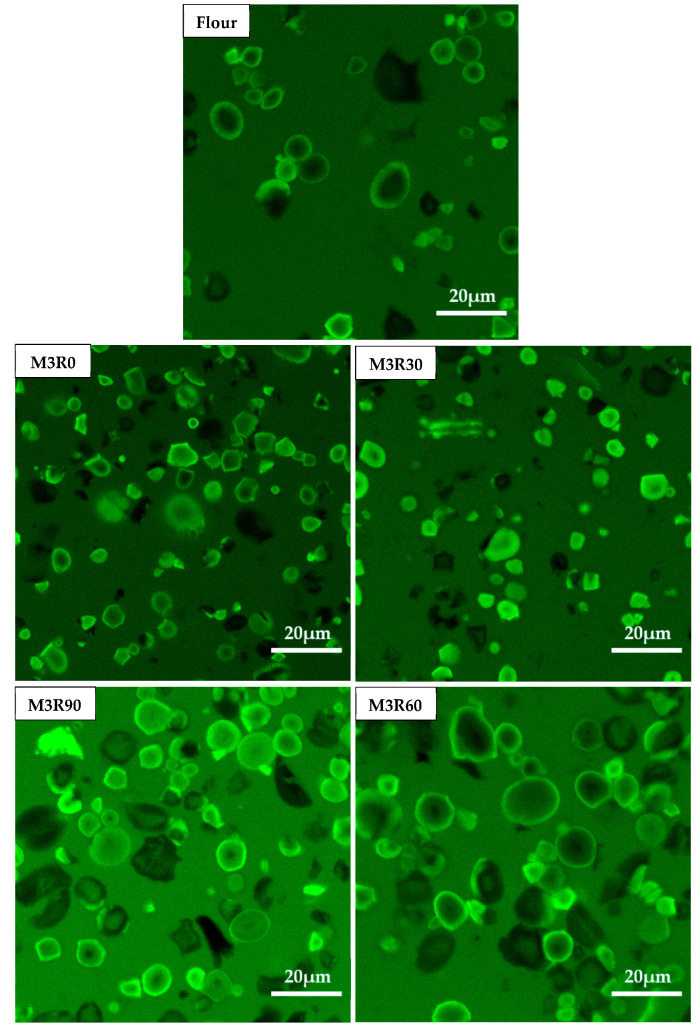

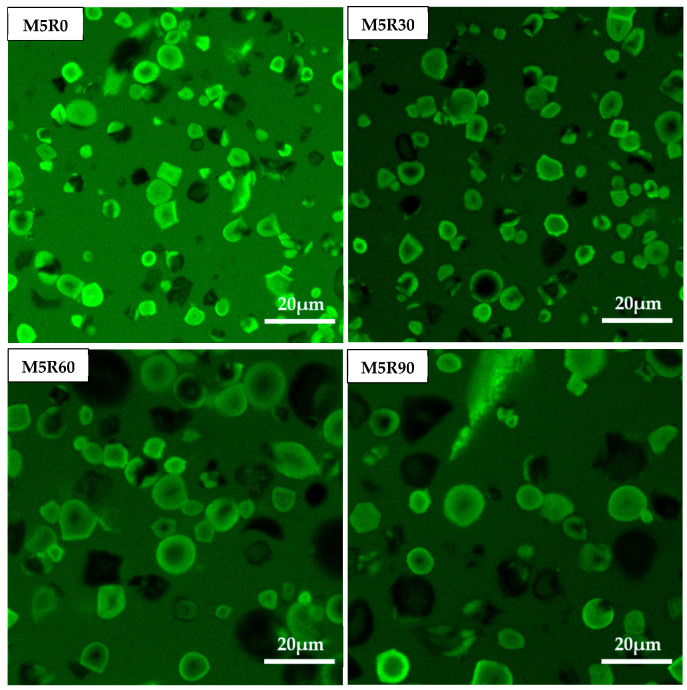
CSLM micrographs of GMP particles. Flour: wheat flour without mixing or resting. M3R0–M3R90: mixing for 3 min and resting for 0, 30, 60, and 90 min, respectively. M5R0–M5R90: mixing for 5 min and resting for 0, 30, 60, and 90 min, respectively.

In this study, CLSM was employed to monitor the microstructure of GMP particles during dough processing, and the results are shown in Figure 6. Fluorescein isothiocyanate (FITC) was selected as the fluorescent dye, which was non-covalently bound to proteins and stained green (the dark areas in Figure 6 are therefore bubbles or the solvent). It is clear from the presented images that the GMP particles in the suspension were spherical or ellipsoidal, and this observation is supported by previously reported results [40]. The GMP particles of the flour group were uniformly ellipsoidal, the particles were intact, and the edges were smooth. However, following mechanical agitation, the GMP particles in the suspension system were no longer regular ellipsoids, but rather displayed irregular edges. For example, the irregular polygons with angular edges are visible in the M3R0 and M5R0 group images. These results indicate that the mechanical force applied during the mixing process changed the protein structure within the GMP particles by affecting the chemical bonds (disulfide bonds) between subunits. Compared with the samples mixed for 3 min, the mechanical agitation for a longer time (5 min) induced more destruction of the GMP particles, leading to a more irregular overall shape of the GMP particles. During the resting process, the GMP particles gradually adopted a more regular ellipsoidal shape and became fuller, with complete and smooth edges, which demonstrates that resting could indeed restore the internal protein structure and chemical bonds. Similarly, it was previously reported [12] that dough mixing induced rearrangement of the dough structure, and dough resting allowed the gluten network structure to be trimmed and rearranged to generate a more continuous and more uniform structure.

However, we observed that some particles still appeared as irregular polygons following the resting process (M3R60, M3R90, M5R60, and M5R60 groups, Figure 6), indicating that the mechanical force during the mixing process irreversibly destroyed some GMP protein particles and they could not fully return to their initial state by resting.

## 3. Materials and Methods

### 3.1. Chemicals and Materials

High-gluten wheat flour (gluten content ≥ 11.5%, Jinshahe Group Co., Ltd., Hebei, China) was purchased from a local supermarket in Beijing, and its composition was 13.1% moisture, 12.2% protein (N × 5.7), 2.0% lipids, 73.8% carbohydrates, glutenin/gliadin ratio of 1.01, and HMW-GS composition of 1, 7 + 8, 5 + 10. All chemicals, solvents, and reagents used in the experiments were of analytical grade or higher and used without further purification.

### 3.2. Dough Sample Preparation (Mixing and Resting)

The prepared dough samples consisted of 200 g flour and 120 g deionized water (the amount of water was determined according to the result of the Mixolab experiment). The flour and water were kneaded using a dough mixer (HMJ-D3826, Shunde Bear Electric Co. Ltd., Shunde, China) for 3 (M3 groups, development time of the flour shown by the Mixolab experiment) or 5 min (M5 groups, before the end of the stability time of the flour shown by the Mixolab experiment). After mixing, the resting stage lasted for 0, 30, 60, or 90 min (M3R0, M3R30, M3R60, and M3R90 groups; M5R0, M5R30, M5R60, and M5R90 groups) at 30 °C, and the dough samples were sealed with a plastic film to limit the drying-out of the dough samples. The dough samples were freeze-dried and then passed through an 80-mesh sieve.

### 3.3. Isolation of GMP

The GMP gel was extracted based on the method presented in [16] with some modification. A dry powder sample (1.4 g) defatted with hexane was suspended evenly in 28 mL of 1.5% SDS (*w*/*v*) and centrifuged at 20,000× *g* for 30 min at 25 °C. The supernatant was decanted and the gel-like layer on top of the starch was collected as the GMP.

### 3.4. GMP Content Measurements

The content of the GMP was determined based on methods reported in Weegels et al. [2] and Aussenac et al. [1] with some modifications. Approximately 0.5 g of freeze-dried powder sample was suspended evenly in 10 mL of 1.5% SDS (*w*/*v*), then oscillated for 1 h at 25 °C, followed by centrifugation at 20,000× *g* for 15 min at room temperature. The supernatant was discarded, and the extraction was repeated one more time. The protein content in the precipitate (i.e., the GMP content) was determined by the Kjeldahl method, using the equation below.

$$\mathrm{GMP}\;\mathrm{content}\;\left(\%\right)\;=\;\frac{\mathrm{GMP}\;\mathrm{content}}{\mathrm{Crude}\;\mathrm{protein}\;\mathrm{content}\;\mathrm{of}\;\mathrm{flour}}\;\times \;100$$

### 3.5. Dynamic Rheology of GMP Gels

Dynamic frequency measurements were performed using a controlled stress–strain rheometer (Discovery DHR-2, TA Instruments, Shanghai, China), using a parallel-plate geometry (*d* = 40 mm). Dynamic rheological measurements were performed according to a previously presented method [29] with some modifications. The GMP gel was placed on the bottom plate. The upper plate was lowered until the plates had a fixed gap of 2 mm and allowed to rest for 5 min after loading to restore the structural damage incurred during the additive process. Methyl silicone oil was used to minimize sample dehydration during the measurement. The G′ and viscosity modulus G″ were measured within the frequency range of 0.01–10 Hz at a constant strain of 0.5% at 25 °C.

### 3.6. Thiol/Disulfide Content in GMP

The accessible thiol (SH_free_), total thiol equivalent group (SH_eq_), and disulfide bond (SS) contents in GMP were determined according to a previously described method [41]. The absorbance of the nitro-thiobenzoate anion was measured at 412 nm (ε = 13,600 L mol^−1^ cm^−1^) using a Cary 100 UV–Vis spectrophotometer (Agilent Technologies Co., Ltd., Santa Clara, CA, USA). The SS content was determined based on the SH_free_ and SH_eq_ contents (SH_eq_ = 2SS + SH_free_).

### 3.7. Particle Size Analysis of GMP

Approximately 1 g of freshly prepared wet GMP sample was added to a 15 mL centrifuge tube and dispersed in 10 mL of 1.5% (*w*/*v*) SDS. After low-speed magnetic stirring for 2 h, the particle size distribution of the GMP was measured using a laser particle size analyzer (SALD-2300N, SHIMADZU, Tokyo, Japan).

### 3.8. Fourier Transform Infrared Spectroscopic Analysis of GMP

Fourier transform infrared (FTIR) spectroscopy (Nicolet iS10, Thermo Fisher Scientific Co. Ltd., Waltham, MA, USA) was used to investigate secondary structures present in the GMP according to a previously described method [28].

### 3.9. HMW/LMW Ratio Determination Statistical Analysis

The HMW/LMW ratios in GMP proteins from flour and dough samples were determined by RP-HPLC according to a previously described method [42] using a Shimadzu HPLC instrument with a C8 stationary phase column. The GMP (100 mg) was extracted with 1 mL 50% (*v*/*v*) 1-propanol, 2 mol/L of urea, 1% (*w*/*v*) dithiothreitol, and 0.05 mol/L of Tris-HCl (pH 7.5) under nitrogen atmosphere. Solvent A for chromatography contained ultra-pure water with 0.1% trifluoroacetic acid (TFA) (*v*/*v*), and solvent B contained acetone (preparative grade) with 0.1% TFA (*v*/*v*). The column was loaded with 100 μL of GMP extract, and the temperature was held constant at 50 °C. The mixture was eluted at a flow rate of 1 mL/min, with a multi-step gradient starting at 28% solvent B and eventually reaching 56% solvent B.

### 3.10. Microstructure of GMP

The microstructure of the GMP was observed using SEM (S-3000N, Hitachi, Tokyo, Japan) according to a previously described method [43].

### 3.11. Confocal Laser Scanning Microscopy

The CLSM measurements of the GMP were conducted according to a previously described method [17].

### 3.12. Statistical Analysis

Data from replicate analyses are presented as the mean value ± standard error (n = 3), and all significance of difference values was obtained using the Tukey test with a confidence level of 95%.

## 4. Conclusions

Different dough processing conditions affect the rheology, amount, secondary structures, SS content, and particle size distribution of the GMP in the dough. Mechanically mixing the dough causes depolymerization of the GMP, resulting in reduced overall GMP content and fewer SS bonds. Additionally, mixing leads to a more disordered protein secondary structure, more irregular protein morphology, and changes to the HMW/LMW subunits ratio. In contrast, resting the dough increases the content of the GMP because of the SS bonds formed gradually during this process, allowing polymerization behavior. The resting process also generally increases the stability and order of the protein secondary structure and pore microstructures. However, resting cannot completely restore the properties of the GMP that are affected by mixing, although it can restore dough properties to some extent. Additionally, excessive resting times may lead to negative results, which manifest in lower GMP and SS contents, and smaller particle size. The study can provide theoretical support and research ideas for the relationship between dough characteristics and GMP changes during processing. In addition, further work is needed to investigate the subunit and amino acid mechanisms responsible for the changes to the GMP during dough mixing and resting. Moreover, different flour cultivars and HMW-GS compositions should be taken into consideration in further concerned research.

## Figures and Tables

**Table 1:** Contents of accessible thiol (SH_free_), total thiol equivalent (SH_eq_), and disulfide bonds (SSs) of the GMP. Data are mean values ± standard variation of three replications and different letters indicate a significant difference at *p* < 0.05.

Samples	SH_free_ (μmol/g)	SH_eq_ (μmol/g)	SS (μmol/g)
Flour	1.44 ± 0.16 ^a^	5.51 ± 0.02 ^f^	2.04 ± 0.07 ^ef^
M3R0	1.36 ± 0.07 ^ab^	5.62 ± 0.01 ^e^	2.13 ± 0.04 ^e^
M3R30	1.13 ± 0.04 ^b^	6.89 ± 0.03 ^c^	2.88 ± 0.02 ^c^
M3R60	1.09 ± 0.13 ^bc^	6.96 ± 0.04 ^b^	2.93 ± 0.07 ^b^
M3R90	1.04 ± 0.13 ^c^	5.43 ± 0.01 ^g^	2.19 ± 0.05 ^e^
M5R0	1.43 ± 0.30 ^a^	5.29 ± 0.03 ^h^	1.91 ± 0.15 ^f^
M5R30	1.21 ± 0.05 ^b^	6.27 ± 0.01 ^c^	2.76 ± 0.02 ^b^
M5R60	1.05 ± 0.03 ^c^	7.08 ± 0.01 ^a^	3.02 ± 0.01 ^a^
M5R90	1.03 ± 0.12 ^c^	6.17 ± 0.01 ^d^	2.55 ± 0.05 ^d^

Flour: wheat flour without mixing or resting. M3R0–M3R90: mixing for 3 min and resting for 0, 30, 60, and 90 min, respectively. M5R0–M5R90: mixing for 5 min and resting for 0, 30, 60, and 90 min, respectively.

**Table 2:** Volume distribution of the GMP (μm) particle. Data are mean values ± standard variation of three replications and different letters indicate a significant difference at *p* < 0.05.

Samples	<11 μm (%)	11–50 μm (%)	>50 μm (%)
Flour	31.12 ± 0.25 ^c^	25.07 ± 0.49 ^a^	43.81 ± 0.55 ^c^
M3R0	33.17 ± 0.18 ^b^	21.57 ± 0.07 ^c^	45.26 ± 0.23 ^b^
M3R30	30.57 ± 0.77 ^cd^	17.30 ± 0.81 ^d^	52.77 ± 1.87 ^ab^
M3R60	29.25 ± 1.23 ^d^	18.66 ± 2.34 ^d^	53.62 ± 2.35 ^a^
M3R90	30.40 ± 0.49 ^cd^	21.77 ± 2.30 ^b^^c^	49.44 ± 4.29 ^b^
M5R0	38.52 ± 0.72 ^a^	22.43 ± 0.46 ^b^	38.26 ± 1.12 ^d^
M5R30	38.34 ± 1.41 ^a^	21.55 ± 0.71 ^c^	39.64 ± 0.70 ^cd^
M5R60	35.02 ± 0.10 ^b^	24.41 ± 0.33 ^a^^b^	40.57 ± 0.38 ^c^
M5R90	37.81 ± 0.51 ^a^	24.82 ± 0.33 ^ab^	37.92 ± 0.82 ^d^

Flour: wheat flour without mixing or resting. M3R0–M3R90: mixing for 3 min and resting for 0, 30, 60, and 90 min, respectively. M5R0–M5R90: mixing for 5 min and resting for 0, 30, 60, and 90 min, respectively.

**Table 3:** Percentage of secondary structures of the GMP. Data are mean values ± standard variation of three replications and different letters indicate a significant difference at *p* < 0.05.

Samples	α-Helix (%)	β-Sheets (%)	β-Turns (%)	Random-Coil (%)
Flour	26.46 ± 1.67 ^d^	32.74 ± 0.44 ^b^	26.75 ± 1.27 ^c^	12.27 ± 0.99 ^a^
M3R0	29.71 ± 0.92 ^bc^	26.87 ± 1.72 ^d^	27.81 ± 1.23 ^b^	11.37 ± 0.01 ^b^
M3R30	29.94 ± 0.28 ^bc^	31.35 ± 0.60 ^c^	27.58 ± 0.27 ^b^	10.85 ± 0.68 ^bc^
M3R60	30.75 ± 0.17 ^b^	32.09 ± 0.57 ^bc^	28.62 ± 0.51 ^b^	10.21 ± 0.72 ^cd^
M3R90	34.24 ± 2.47 ^a^	33.02 ± 0.32 ^ab^	29.68 ± 1.91 ^a^	8.49 ± 0.67 ^f^
M5R0	28.58 ± 0.13 ^c^	32.26 ± 0.01 ^bc^	28.34 ± 0.31 ^b^	10.26 ± 0.58 ^cd^
M5R30	29.31 ± 0.10 ^b^	32.50 ± 0.10 ^bc^	28.71 ± 0.15 ^b^	10.06 ± 0.04 ^d^
M5R60	29.39 ± 0.47 ^bc^	32.65 ± 0.14 ^bc^	28.79 ± 0.24 ^b^	9.78 ± 0.40 ^e^
M5R90	31.05 ± 0.81 ^b^	33.92 ± 1.00 ^a^	28.43 ± 1.55 ^b^	10.10 ± 0.02 ^d^

Flour: wheat flour without mixing or resting. M3R0–M3R90: mixing for 3 min and resting for 0, 30, 60, and 90 min, respectively. M5R0–M5R90: mixing for 5 min and resting for 0, 30, 60, and 90 min, respectively.

## Data Availability

The data presented in this study are available on request from the corresponding author.
